# Corneal neovascularization and biological therapy

**Published:** 2015

**Authors:** OB Voiculescu, LM Voinea, C Alexandrescu

**Affiliations:** * Department of Cellular and Molecular Medicine, ”Carol Davila” University of Medicine and Pharmacy, Bucharest, Romania; ** Department of Ophthalmology, University Emergency Hospital Bucharest, Romania

**Keywords:** corneal neovascularization, vascular endothelial growth factor, anti VEGF therapy

## Abstract

Corneal avascularity is necessary for the preservation of optimal vision. The cornea maintains a dynamic balance between pro- and antiangiogenic factors that allows it to remain avascular under normal homeostatic conditions. Corneal neovascularization (NV) is a condition that can develop in response to inflammation, hypoxia, trauma, or limbal stem cell deficiency and it is a significant cause of blindness. New therapeutic options for diseases of the cornea and ocular surface are now being explored in experimental animals and clinical trials. Antibody based biologics are being tested for their ability to reduce blood and lymphatic vessel ingrowth into the cornea, and to reduce inflammation. Numerous studies have shown that biologics with specificity for VEGF A such as bevacizumab and ranibizumab (a recombinant antibody and an antibody fragment, respectively) or anti-tumor necrosis factor-α microantibody, are effective in the treatment of corneal neovascularization.

## Introduction 

A healthy cornea is necessary to provide a proper anterior refractive surface and to protect the eye against infection and structural damage to the deeper components of the eye. Corneal transparency and optimal vision require an avascular cornea. Maintaining the avascularity of the corneal stroma is an important aspect of the corneal pathophysiology. Blood vessels are present in all mesenchymal or connective tissues, except for cartilage and the corneal stroma. The establishment and maintenance of an avascular stroma is an important aspect of the corneal development and physiology.

Diseases associated with corneal NV include inflammatory disorders, corneal graft rejection, infectious keratitis, contact lens–related hypoxia, alkali burns, stromal ulceration, aniridia, and limbal stem cell deficiency [**1**]. Neovascularization may invade the cornea at deeper levels depending on the nature and location of the inflammatory stimulus. The normally avascular cornea may vascularize in situations in which a disequilibrium between angiogenic and antiangiogenic stimuli lead to a surplus of pro-angiogenic factors, such as vascular endothelial growth factor [VEGF], basic fibroblast growth factor [bFGF], and matrix metalloproteinases and a deficiency in antiangiogenic factors, pigment epithelium–derived factor, angiostatin and endostatin [**2**].

Current treatments for corneal neovascularization include topical corticosteroid and non-steroid anti-inflammatory medications, photodynamic therapy, laser photocoagulation, fine needle diathermy, and conjunctival, limbal, and amniotic membrane transplantation. Unfortunately, all these have a limited clinical efficacy and also cause a multitude of undesirable side effects, especially elevated intraocular pressure and posterior subcapsular cataracts subsequent to corticosteroid use.

**Vascular endothelial growth factor**

Vascular endothelial growth factor (VEGF) has a prominent role in the physiological and pathological angiogenesis. Physiological VEGF expression is now known to be important for protection of hepatocytes and renal cells, for wound healing, female reproductive cycling, bone growth, trophic maintenance of capillaries and neurons. In the eye, VEGF plays a physiological role in the development and trophic maintenance of the choriocapillaris and in protecting retinal neurons from apoptosis in conditions of ischaemia [**7**].

Vascular endothelial growth factor (VEGF) plays a key role in vasculogenesis and the pathologic neovascularization (NV) associated with eye disease. Although anti-VEGF therapy for ocular disease has been principally directed at the retinal vascular conditions, it is widely accepted that anti-VEGF therapy is also effective when used to treat corneal NV [**25**].

VEGF (also known as VEGF-A) is a secreted growth factor peptide that belongs to a gene family that includes VEGF-B, VEGF-C, VEGF-D, VEGF-E, and placental growth factor (PlGF). VEGF-A is the main regulator of hemangiogenesis, whereas VEGF-C and VEGF-D are key regulators of lymphangiogenesis [**3**]. Overproduction of VEGF-A was observed in tumor cell proliferation, similarly to corneal neovascularization formation. VEGF-A sustains several steps of angiogenesis including proteolytic activity, vascular endothelial cell proliferation, migration and capillary lumen formation. The importance of VEGF-A in corneal angiogenesis was demonstrated experimentally on animal models by inhibiting neovascularization after stromal application of an anti-VEGF-A antibody [**10**].

VEGF promotes vascular endothelial cell proliferation, migration, and tube formation [**4**]. It also increases vascular leakage and promotes monocyte chemotaxis and B-cell production in mice, indicating the key role of VEGF in inflammation [**5**].

The four known isoforms of VEGF bind to tyrosine kinase receptors on vascular endothelial cells, causing their division and migration. Two VEGF receptors belonging to the tyrosine-kinase receptor family have been identified and cloned: the VEGFR-1 and the VEGFR-2 receptors. Along with the VEGFR-3 receptor, which is expressed in lymph vessels and binds VEGF-C and VEGF-D, these receptors form a subfamily distinguished by the presence of seven immunoglobulin-like loops in their extracellular part and a split tyrosine-kinase domain in their intracellular part. The VEGFR-2 and VEGFR-1 receptors are predominantly expressed in endothelial cells, but a few additional types of cells express one or both of these receptors. The VEGFR-1 receptor is expressed in trophoblast cells, monocytes, and renal mesangial cells. VEGFR-2 is expressed in hematopoietic stem cells, megakaryocytes, and retinal progenitor cells [**6**]. 

**Anti VEGF antibody**

One possible strategy for treating corneal neovascularization is to inhibit VEGF activity by competitively binding VEGF with a specific neutralizing anti-VEGFantibody. Anti-VEGF agents have demonstrated efficacy in reducing corneal neovascularization in both animal models and clinical trials. VEGF inhibitors such as pegaptanib sodium (Macugen™, OSI/Eyetech), ranibizumab (Lucentis™, Genentech) and off-label bevacizumab (Avastin™, Genentech) are currently used for the treatment of various retinal diseases such as neovascular age-related macular degeneration [**16**]. 

**Bevacizumab** is a full-length, humanized murine monoclonal antibody with a molecular weight of 149kD that recognizes all isoforms of VEGF. Bevacizumab is FDA-approved for intravenous administration in the treatment of several cancers and is in widespread use, off-label, as an intravitreal injection to treat a variety of retinal pathologies [**8**,**9**]. In addition, studies have exhibited a partial reduction of corneal neovascularization through topical, subconjunctival, and intraocular application of bevacizumab and improving corneal transparency following corneal alkali burn injury by accelerating regeneration of the basement membrane [**12**,**13**]. 

Subconjunctival administration of bevacizumab induced an involution of new vessels, abolished inflammatory response and resulted in return of corneal function [**14**]. Studies demonstrated that bevacizumab is nontoxic to human corneal epithelial and fibroblast cells [**15**]. Bevacizumab effectively inhibits corneal NV only if administered early because chronic NV may contain matured vessels covered with pericytes and smooth muscle cells. In the absence of pericytes and smooth muscle cells coating, NV may regress when angiogenic factors are downregulated. Bevacizumab could inhibit macrophage infiltration in early but not late treatment groups. Macrophages are known to trigger NV in inflamed or ischemic tissues including the cornea,and bevacizumab has been reported to inhibit NV formation in inflamed or hypoxic corneal tissues [**24**]. 

Tacrolimus is an immunosuppressive drug mainly used after allogeneic organ transplant to reduce the activity of the patient’s immune system and to lower the risk of organ rejection. It is also used in a topical preparation in the treatment of atopic dermatitis (eczema), severe refractory uveitis after bone marrow transplants. 

Comparative studies between bevacizumab and tacrolimus show that topical and subconjunctival tacrolimus application may be useful in reducing corneal NV and have comparable effects to subconjunctival bevacizumab injection [**21**].

The topical administration of both bevacizumab and sunitinib (anti VEGF and anti PDGF) inhibits corneal neovascularization in rabbits. But vascular endothelial growth factor (VEGF) pathway blockade by bevacizumab is not sufficient for a profound inhibition. Blocking both VEGF and platelet-derived growth factor pathways by using sunitinib is three times more effective [**11**].

**Tocilizumab** is a humanized monoclonal antibody against the interleukin-6 receptor (IL-6R). Interleukin 6 (IL-6) is a cytokine that plays an important role in immune response and is implicated in the pathogenesis of many diseases, such as autoimmune diseases, multiple myeloma and prostate cancer. An antiangiogenic effect was observed after subconjunctival injection of 2.5 mg tocilizumab to an extent similar to that seen with 2.5 mg bevacizumab, which indicates that subconjunctival application of tocilizumab is effective for the inhibition of corneal neovascularization [**22**].

**Ranibizumab** is a recombinant humanized monoclonal antibody fragment that binds and inhibits VEGF-A isoforms. Ranibizumab has a molecular weight of 48kD, making it approximately one-third the size of bevacizumab and theoretically allowing a better corneal penetration; additionally, ranibizumab has been affinity-matured to optimize its VEGF-A binding potential. These characteristics may enable ranibizumab to treat corneal NV more effectively than bevacizumab [**20**].

Bevacizumab and ranibizumab have similar VEGF-binding characteristics because both drugs are related to each other. Ranibizumab is the Fab fragment from the same antibody used to create bevacizumab, but it has been affinity-matured so that it binds VEGF-A with significantly higher affinity. Topical ranibizumab 1% is effective in the treatment of clinically stable corneal NV as evidenced by a significant reduction in two corneal NV parameters (neovascular area and vessel caliber). The significant reduction of neovascular area and vessel caliber in the absence of a significant change in invasion area suggests that the main outcome of ranibizumab treatment is to induce the narrowing of blood vessels more than a reduction in their length. Stable NV is less influenced by VEGF blockade as opposed to newly formed vessels; this may explain the absence of significant reduction in the NV invasion area [**17**]. Experimental studies on rabbits demonstrated that early subconjunctival administration of ranibizumab may successfully inhibit alkali-induced corneal neovascularization. Subconjunctival ranibizumab reduces VEGF levels significantly not only in the cornea and the bulbar conjunctiva but also in the aqueous humor and the iris [**18**]. A comparative study of ranibizumab and bevacizumab on corneal neovascularization in rabbits indicated that subconjunctival administrations of both substances inhibit corneal NV in rabbits and have equivalent effects on it [**19**].

The effect of the anti-VEGF agents was dependent on time of the treatment after the onset of NV. Early subconjunctival bevacizumab administration (one day after injury) inhibited corneal NV more effectively in a rabbit experimental model of limbal insufficiency than when treatment was performed on day 14. Recently, Lin et al. [**26**] demonstrated that the earlier the time of treatment with subconjunctival bevacizumab, the better the result in rabbit eyes with limbal insufficiency. Early and mid-point bevacizumab injections inhibited epithelial alteration, the development of stromal opacity, and corneal NV manifestation as limbal insufficiency, while the late treatment had no inhibitory effect. The vessels may mature in chronic NV, and pericytes may be recruited to the region around the area of pathologic NV [**27**]. Such coverage may decrease the effect of bevacizumab on the regression of newly formed immature vessels. 

**Pegaptanib** sodium is an RNA aptamer directed against vascular endothelial growth factor (VEGF)-165, the VEGF isoform being primarily responsible for pathological ocular neovascularization and vascular permeability. After nearly a decade of preclinical development to optimize and characterize its biological effects, pegaptanib was shown to be effective in clinical trials in treating choroidal neovascularization associated with age-related macular degeneration. Therefore, Pegaptanib has the notable distinction of being the first aptamer therapeutic approved for use in humans, paving the way for future aptamer applications [**30**].

Subconjunctival bevacizumab, ranibizumab, and pegaptanib sodium is effective with no epitheliopathy in controlling corneal neovascularization after corneal burn in rats. In addition, bevacizumab is more effective than ranibizumab and pegaptanib sodium. Ranibizumab and pegaptanib sodium had a similar efficacy. To improve the effectiveness of treatments, combination therapy with other antiangiogenic agents, and/ or repeated subconjunctival injections with higher doses and concentrations of a longer duration may be valid options [**29**]. 

**Tyrosine kinase inhibitors**

Regorafenib is a multikinase inhibitor that targets kinases, including VEGF receptors 1, 2, and 3; TIE2; platelet-derived growth factor β; mutant oncogenic kinases; and the fibroblast growth factor receptor, which is involved in NV and oncogenesis. Tyrosine kinase with immunoglobulin and epidermal growth factor homology domain 2 (TIE2) is a crucial regulator of angiogenesis that is exclusively or predominantly expressed in endothelial cells. Regorafenib is a novel, potent inhibitor of the VEGF receptor and other angiogenic receptor tyrosine kinases and has inhibitory effects on alkali-induced corneal NV in rats. The inhibitory effects of topical regorafenib were comparable to those of topical dexamethasone and bevacizumab [**23**]. Monoclonal antibodies induce their effect by blocking the effect of VEGF prior to its attachment to the endothelial receptors. Tyrosine kinase inhibitors block the VEGF activity by inhibiting tyrosine kinase in the intracellular part of the VEGF cell membrane receptor. This may represent a different opportunity for the treatment of the neovascular process in ocular pathologies. 

Lapatinib used in the form of lapatinib ditosylate, is an orally active drug for breast cancer and other solid tumours. It is a dual tyrosine kinase inhibitor which interrupts the HER2/ neu and epidermal growth factor receptor (EGFR) pathways. Trastuzumab is a monoclonal antibody that interferes with the HER2/ neu receptor. In recent studies, both substances were compared for the treatment of experimental corneal neovascularization and suggested that systemically administered lapatinib is more effective than systemically administered trastuzumab in preventing corneal neovascularization [**31**].

Antibody based biologics with specificity for tumor necrosis factor-alpha (TNFα) are also currently examined for efficacy in modulating corneal disease in experimental animals. Topical infilximab has been shown to reduce corneal perforation, opacity, infiltration by leukocytes, neovascularization and lymphogenesis folowing a chemical burn [**32**].

## Discussion

VEGF is one of the major factors involved in the pathogenesis of corneal neovascularization. VEGF-A is believed to be the most important member of this family, especially relating to pathologic hemangiogenesis. As a result of variations in mRNA splicing there are four different forms with various numbers of amino acids: VEGF-A121, VEGF-A165, VEGF-A189, and VEGF-A206. VEGF-A165 is the dominant proangiogenic isoform. Both bevacizumab and ranibizumab use the same mechanisms and all inhibit the VEGF-A isoforms nonspecifically [**28**]. Nevertheless, differently from bevacizumab and ranibizumab, pegaptanib does not inhibit all of the VEGF isoforms; it specifically binds to VEGF-A165.

Treating corneal NV with the anti-VEGF antibody does have some limits. It is only a symptomatic treatment of corneal NV that does not cure the cause of the disorder and in some cases it is necessary to repeat the treatment to maintain its positive effect over a period of time. In addition, its effect on deep vascularization is lower in contrast to superficial and active vascularization, in which clear regression is observed [**10**].

The time of starting the treatment might affect the efficacy; beginning the treatment just after the chemical cauterization might cause less corneal neovascularization and the affinity of anti-VEGF agents for VEGF in rats may be lower than in human.

Anti-VEGF agents have generated an enormous hope for the treatment of corneal neovascularization. Large, randomized, controlled clinical trials are essential for the justification of the continued development of these agents. The establishment of safe doses and methods of administration are required before these agents can be used in the clinical setting. Therefore, further investigation is needed before anti-VEGF agents can become key therapeutic agents in the inhibition of corneal angiogenesis.

**Acknowledgement**

This paper was supported by the Sectoral Operational Programme Human Resources Development financed from the European Social Fund under the contract number POSDRU/159/1.5/S/137390.
